# Complexity Reduction in the Use of Evolutionary Algorithms to Function Optimization: A Variable Reduction Strategy

**DOI:** 10.1155/2013/172193

**Published:** 2013-10-23

**Authors:** Guohua Wu, Witold Pedrycz, Haifeng Li, Dishan Qiu, Manhao Ma, Jin Liu

**Affiliations:** ^1^Science and Technology on Information Systems Engineering Laboratory, National University of Defense Technology, 47 Yanzheng Street, Changsha, Hunan 410073, China; ^2^Department of Electrical & Computer Engineering, University of Alberta, Edmonton, AB, Canada T6R 2V4; ^3^Warsaw School of Information Technology, Newelska 6, Warsaw, Poland; ^4^School of Civil Engineering and Architecture, Central South University, Changsha, Hunan 410004, China; ^5^School of Geosciences and Info-Physics, Central South University, Changsha, Hunan, China

## Abstract

Discovering and utilizing problem domain knowledge is a promising direction towards improving the efficiency of evolutionary algorithms (EAs) when solving optimization problems. We propose a knowledge-based variable reduction strategy (VRS) that can be integrated into EAs to solve unconstrained and first-order derivative optimization functions more efficiently. VRS originates from the knowledge that, in an unconstrained and first-order derivative optimization function, the optimal solution locates in a local extreme point at which the partial derivative over each variable equals zero. Through this collective of partial derivative equations, some quantitative relations among different variables can be obtained. These variable relations have to be satisfied in the optimal solution. With the use of such relations, VRS could reduce the number of variables and shrink the solution space when using EAs to deal with the optimization function, thus improving the optimizing speed and quality. When we apply VRS to optimization problems, we just need to modify the calculation approach of the objective function. Therefore, practically, it can be integrated with any EA. In this study, VRS is combined with particle swarm optimization variants and tested on several benchmark optimization functions and a real-world optimization problem. Computational results and comparative study demonstrate the effectiveness of VRS.

## 1. Introduction

Optimization, including continuous optimization and discrete optimization, plays an important role in scientific research, management, industry, and so forth, given the fact that many problems in the real world are essentially optimization tasks. Evolutionary algorithms (EAs), such as genetic algorithms (GAs), ant colony optimization (ACO), and particle swarm optimization (PSO), have shown competitive performance when solving complex and large-scale optimization problems. To improve the efficiency of EAs, two aspects are important and deserve investigation. The first one is the search capability of the used EA itself, including both the exploitation and exploration capabilities. The other one is how to effectively integrate domain knowledge about the optimization problem into EAs [1]. 

Previously, more attention was paid to the design of generic EA variants for higher search capability. Take PSO as an example. In the last decades, many enhanced PSO versions were developed, such as comprehensive learning PSO [2], mimetic fitness Euclidean-distance PSO [3], orthogonal learning PSO [4], and PSO with local search [5]. According to no free lunch theory [6], no algorithm will be effective for all optimization problems. It is therefore hard to design an efficient EA that is suitable to all kinds of optimization problems. However, if we can make use of some valuable domain knowledge implied in optimization problems, we may improve the efficiency of EAs by reducing the complexity of the optimization problems.

In the area of discrete optimization, the problem domain knowledge has started to attract researchers' attention. For instance, the incorporation of knowledge-based strategies into the heuristics of swarm optimization has demonstrated to be effective [7]. Note that the problem domain knowledge in discrete optimization (e.g., the scheduling problem [8, 9] and spatial geoinformation services composition problem [10, 11]) is dependent on concrete problems considered and the knowledge extraction and discovery process is relatively subjective. 

In comparison, in the area of continuous optimization, such as function optimization, the problem domain knowledge is seldom considered. One may consider the integration of PSO with the gradient-based search technique as an instance that the problem domain knowledge is combined into EAs [12], as the gradient-based search technique utilizes the gradient information implied in the optimization problem. This gradient-based search technique is used to guide the search direction of EAs. It is believed that there should be some relations among different variables on the optima of an optimization problem. Particularly, in [1], the problem domain knowledge of variable symmetry was formulated. Based on this knowledge, an inner variable learning (IVL) strategy was proposed and incorporated into PSO, thus a new PSO variant named PSO-IVL was developed. PSO-IVL demonstrates the effectiveness and potential of the integration of problems domain knowledge into EAs. However, it is not a generic algorithm because it is only suitable to optimization problems with symmetric variables. 

Therefore, we may wonder whether there exist more general methods for finding relations among different variables of an optimization problem; then we can utilize the knowledge of such relations to simplify the original optimization problems and improve the efficiency of EAs. As we know, the variable relation reflects the variable dependence, which can shrink the solution space of original optimization problems.

Driven by this motivation, we investigate a method to discover underlying variable relations existing in an unconstrained and first-order derivative optimization function. We find that the discovered variable relations can be used effectively to reduce the number of variables included in the optimization function when applying PSO (other EAs should be suitable as well) to that function optimization problem. Consequently a variable reduction strategy (VRS) is developed and integrated into the PSO variants. Experimental tests on some benchmark optimization functions and a real-world optimization problem demonstrate that VRS can reduce the complexity of the optimization functions and help PSO to find high-quality solutions more efficiently. 

## 2. Variable Reduction Strategy

Assume that *f*(*x*_1_, *x*_2_,…, *x*_*D*_) has a first-order derivative. For the corresponding unconstrained optimization problem min⁡*f*(*x*_1_, *x*_2_,…, *x*_*D*_), the optimal solution arises from the relationships

(1) ∂f(x1,x2,…,xD)∂x1=0,∂f(x1,x2,…,xD)∂x2=0,    ⋮∂f(x1,x2,…,xD)∂xD=0.

It may sometimes be difficult to solve the equations above to obtain exact values of their variables. There are two reasons for this. The first one is that the equations may be nonlinear and complex which makes it difficult to get the completely analytic solution. The second one is that there might be many extreme points for a multimodal optimization function; that is to say, solutions of the equations related to such an optimization function are not unique. However, some quantitative and explicit relations among variables could be determined from (1). We do not have to find out all the relations among variables, since if we can obtain just some variable relations from (1), we can reduce the number of variables and shrink the solution space, thus, decreasing the complexity of the original optimization function. 

For example, if from (1) we can form a relation described as

(2) $$\begin{array}{c} x_{i}=g\left(\left\{x_{j}\;|\;i=1,\ldots ,m,\;j\neq i\right\}\right),\;1\leq i\leq D,\;1\leq m\leq D, \end{array}$$

we say *x*_*i*_ can be expressed by {*x*_*j*_ | *i* = 1,…, *m*,  *j* ≠ *i*}. This variable relation has to be satisfied in the optimal solution. Under this condition, in the course of using EA (e.g., PSO) to solve the optimization function, the value of *x*_*i*_ can be calculated directly from (2) and the values of variables in {*x*_*j*_ | *i* = 1,…, *m*,  *j* ≠ *i*}. As a result, in the problem solving process, variable *x*_*i*_ can be reduced. 

**Figure 1:**
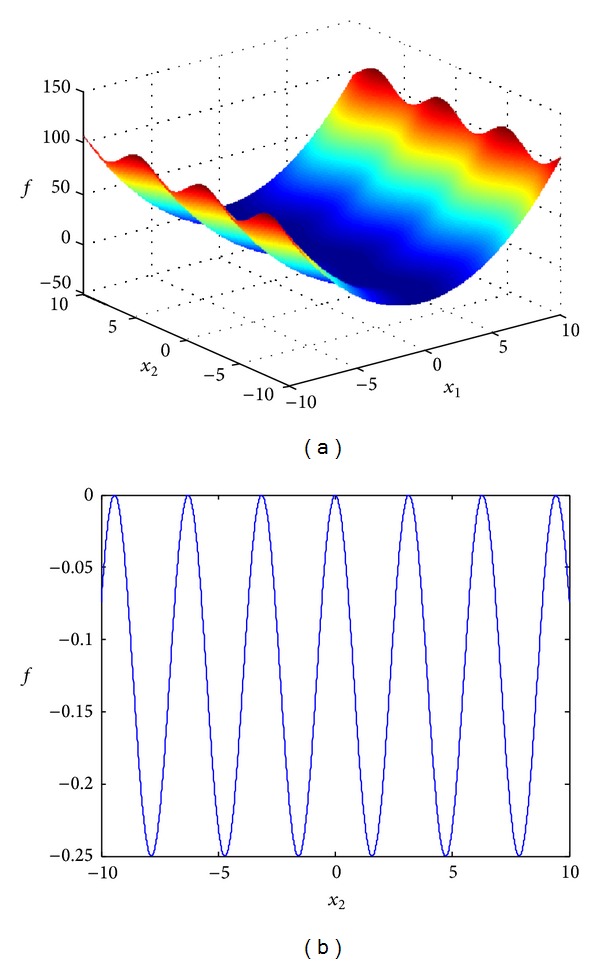
Illustration of an example of variable reduction strategy. (a) Solution space of the original problem; (b) solution space of the problem after variable reduction.

To give an intuitive illustration, let us now consider optimization problem min⁡*f* = *x*_1_^2^ + *x*_1_ · sin⁡*x*_2_ as an example. The solution space of this optimization problem is illustrated in Figure 1(a). Let the derivative of the optimization function be equal to zero; we can obtain the following:

(3) $$\begin{array}{c} 2x_{1}+\sin x_{2}=0, \end{array}$$

(4) $$\begin{array}{c} x_{1}\cdot \cos x_{2}=0. \end{array}$$

From (3), we get the relation *x*_1_ = −0.5sin*x*_2_. With this relation, variable *x*_1_ can be reduced and the original optimization problem is changed into min⁡*f* = −0.25(sin*x*_2_)^2^. The solution space of the optimization problem after variable reduction is changed accordingly as displayed in Figure 1(b). As a result, the original two-variable optimization problem is transformed into a one-variable optimization problem after variable reduction. In addition, the solution space is shrunk from two dimensions to one dimension. Therefore, with variable reduction, the complexity of this optimization problem is reduced noticeably. 

It is clear that if more variable relations can be found, then more variables can be reduced. Let us introduce several essential definitions.

- Core variable: the variable is used to represent other variables.
- Reduced variable: the reduced variable can be represented by core variables.
- Optimization variable core: the collection of all core variables present in an optimization function. We denote it by *C* = {*x*_*i*_ | *i* = 1,…, *n*}, 1 ≤ *n* ≤ *D*.

Obviously, the fewer core variables and the more reduced variables we obtain, the more the complexity of the original optimization function will be eliminated. Therefore, the task of the variable reduction strategy (VRS) is to obtain an optimization variable core with the minimum cardinality. To obtain a general theory and method for finding minimum core variables is still an open problem, since the equations described in (1) may be too complex. However, at least, we can safely conclude that if a variable in a derivative optimization problem is less than or equal to third-order, and this variable can be reduced.

In general, the performance of EAs degrades noticeably with the dimensionality increase of an optimization problem. VRS can help alleviate the problem.

## 3. Experimental Study on Benchmark Optimization Problems

### 3.1. Experimental Setting

VRS is integrated into the basic version of PSO [13] to obtain a new PSO variant called PSO-VRS. We apply PSO-VRS to several benchmark optimization functions to test the effectiveness of VRS. The Rosenbrock function [2], variably dimensioned function [14], Wood function [14], and Ackley function [2] are selected as the benchmark optimization functions. Each function was optimized by some state-of-the-art PSO variants as well as PSO-VRS. We present the details of the variable reduction procedure for each function, provide the computational results of each algorithm, and demonstrate the evolutionary process of PSO-VRS. The PSO variants used in the comparative study are listed below:

- PSO with inertia weight (PSO-w) [15];
- PSO with constriction factor (PSO-cf) [16];
- UPSO [17];
- fully informed particle swarm (FIPS) [18];
- FDR-PSO [19];
- CPSO-H [20];
- CLPSO [2];
- PSO-VRS.

It should be noted that the parameter settings of PSO-w, PSO-cf, UPSO, FDR, FIPS, CPSO-H, and CLPSO are the same as those in [2]. Related parameters of PSO-VRS are set up as follows: the inertia weight *w* = 0.9 − (0.5 · *g*/max⁡⁡Gen), the maximum function evaluations max⁡⁡FEs = 200000, acceleration coefficients *c*_1_ = *c*_2_ = 2.0, and number of particles *ps* = 20. 

### 3.2. Variable Reduction Process of Test Optimization Functions

#### 3.2.1. Rosenbrock Function

This function is formulated as *f*(**x**) = ∑_*i*=1_^*D*^(100(*x*_*i*_^2^ − *x*_*i*+1_)^2^ + (*x*_*i*_ − 1)^2^), which is multimodal, is nonseparable, and exhibits a very narrow valley moving from local optimum to global optimum [21]. Let us rewrite the function as follows:

(5) f(x)=∑i=1n−1(100(xi2−xi+1)2+(xi−1)2)=100(x12−x2)2+(x1−1)2+100(x22−x3)2+(x2−1)2+100(x32−x4)2+(x3−1)2+⋯+100(xn−12−xn)2+(xn−1−1)2.

Then setting the partial derivatives to zero, we have

(6) $$\begin{array}{c} \frac{df}{dx_{1}}=200\left(x_{1}^{2}-x_{2}\right)\cdot 2x_{1}+2\left(x_{1}-1\right)=0, \end{array}$$

(7) dfdxi=−200(xi−12−xi)+200(xi2−xi+1)·2xi+2(xi−1)=0, 2<i<n,

(8) $$\begin{array}{c} \frac{df}{dx_{n}}=-200\left(x_{n-1}^{2}-x_{n}\right)=0. \end{array}$$

From (6), we have

(9) $$\begin{array}{c} x_{2}=x_{1}^{2}+\frac{x_{1}-1}{200x_{1}}. \end{array}$$

Subsequently from (7), we have

(10) $$\begin{array}{c} x_{i}=x_{i-1}^{2}+\frac{\left(x_{i-1}-1\right)-100\left(x_{i-2}^{2}-x_{i-1}\right)}{200x_{i-1}},\;2<i<n. \end{array}$$

Furthermore,

(11) $$\begin{array}{c} x_{n}=x_{n-1}^{2}. \end{array}$$

We can observe from the above expressions (9)–(11) that any other variables in the Rosenbrock function can be calculated with the use of *x*_1_, such that the objective function can be calculated only with the aid of the value of *x*_1_ and (9)–(11). Therefore *x*_1_ is the one and only one core variable of this optimization function. As a result, the original multivariable optimization problem is actually transformed into a one-variable optimization problem. The Rosenbrock function with 10 variables was optimized by each PSO variant. The search range of each variable is taken from −3 to 3. 

#### 3.2.2. Variably Dimensioned Function

This function is described as *f*(*x*) = ∑_*i*=1_^*n*^(*x*_*i*_ − 1)^2^ + (∑_*i*=1_^*n*^ *i*(*x*_*i*_ − 1))^2^ + (∑_*i*=1_^*n*^ *i*(*x*_*i*_ − 1))^4^. With regard to this function, we obtain

(12) dfdxi=2(xi−1)+2i(∑i=1ni(xi−1))+4i(∑i=1ni(xi−1))3=0, 1≤i≤n.

According to (12), for any two variables *x*_*i*_ and *x*_*k*_ we have

(13) $$\begin{array}{c} 2\left(x_{i}-1\right)+2i\left(\overset{n}{\underset{i=1}{\sum }}i\left(x_{i}-1\right)\right)=-4i{\left(\overset{n}{\underset{i=1}{\sum }}i\left(x_{i}-1\right)\right)}^{3},\\ 2\left(x_{k}-1\right)+2k\left(\overset{n}{\underset{i=1}{\sum }}i\left(x_{i}-1\right)\right)=-4k{\left(\overset{n}{\underset{i=1}{\sum }}i\left(x_{i}-1\right)\right)}^{3}. \end{array}$$

From (13), we get

(14) $$\begin{array}{c} k\left(x_{i}-1\right)=i\left(x_{k}-1\right). \end{array}$$

In the sequel, from (14), we can obtain *x*_*i*_ = (*ix*_*k*_ − *i* + *k*)/*k*.

Let *k* = 1, then we have *x*_*i*_ = *ix*_1_ − *i* + 1. As a result all other variables can be represented by *x*_1_, which is the core variable of this optimization function.

#### 3.2.3. Wood Function

This function comes in the form

(15) f(x)=(10(x2−x12))2+(1−x1)2+((90)1/2(x4−x32))2+(1−x3)2+((10)1/2(x2+x4−2))2+((10)−1/2(x2−x4))2.

We determine the related derivative as shown below:

(16) dfdx1=200(x2−x12)(−2x1)−2(1−x1)=0,dfdx2=200(x2−x12)+20.2x2+19.8x4−40=0,dfdx3=180(x4−x32)(−2x3)−2(1−x3)=0,dfdx4=180(x4−x32)+19.8x2+20.2x4−40=0.

From (16), one has

(17) x2=x1−1200x1+x12,

(18) x4=1000(x2−x12)+101x2−200−99,

(19) x4=x3−1180x3+x32,

(20) x3=1001x4+99x2−200900.

We can see from (17)–(20) that *x*_2_ can be calculated from *x*_1_; *x*_4_ can be calculated with the use of both *x*_2_ and *x*_1_; and *x*_3_ can be calculated based on *x*_4_ and *x*_2_. In fact, all *x*_4_, *x*_3_, and *x*_2_ can be computed from *x*_1_, which is therefore the only core variable of this optimization function. Note that, in the search process of PSO-VRS, the value of (1001*x*_4_ + 99*x*_2_ − 200)/900 in (20) may be smaller than zero. Under this condition, the function fitness will be penalized and equal to 1000, to drive the solution to the feasible area.

#### 3.2.4. Ackley Function

This function reads as $f_{5}\left(x\right)=-20\cdot \exp\left(-0.2\sqrt{(1/n)\sum_{i=1}^{n}x_{i}^{2}}\right)+20-\exp\left((1/n)\sum_{i=1}^{n}\cos\left(2\pi x_{i}\right)\right)+e$.

Regarding this function, the partial derivatives are

(21) dfdxj=−20exp⁡(−0.21n∑i=1nxi2)×(−0.2)121(1/n)∑i=1nxi21n2xj−exp⁡(1n∑i=1ncos⁡⁡(2πxi))1n2πsin(2πxj)=0,dfdxk=−20exp⁡(−0.21n∑i=1nxi2)×(−0.2)121(1/n)∑i=1nxi21n2xk−exp⁡(1n∑i=1ncos⁡⁡(2πxi))1n2πsin(2πxk)=0.

From (21), we obtain the following relationship:

(22) $$\begin{array}{c} \frac{x_{j}}{x_{k}}=\frac{\sin\left(2\pi x_{j}\right)}{\sin\left(2\pi x_{k}\right)}. \end{array}$$

Such that we have *x*_*j*_ = *x*_*k*_. Therefore, variable *x*_1_ can be the only core variable.

### 3.3. Computational Results and Comparative Study

**Figure 2:**
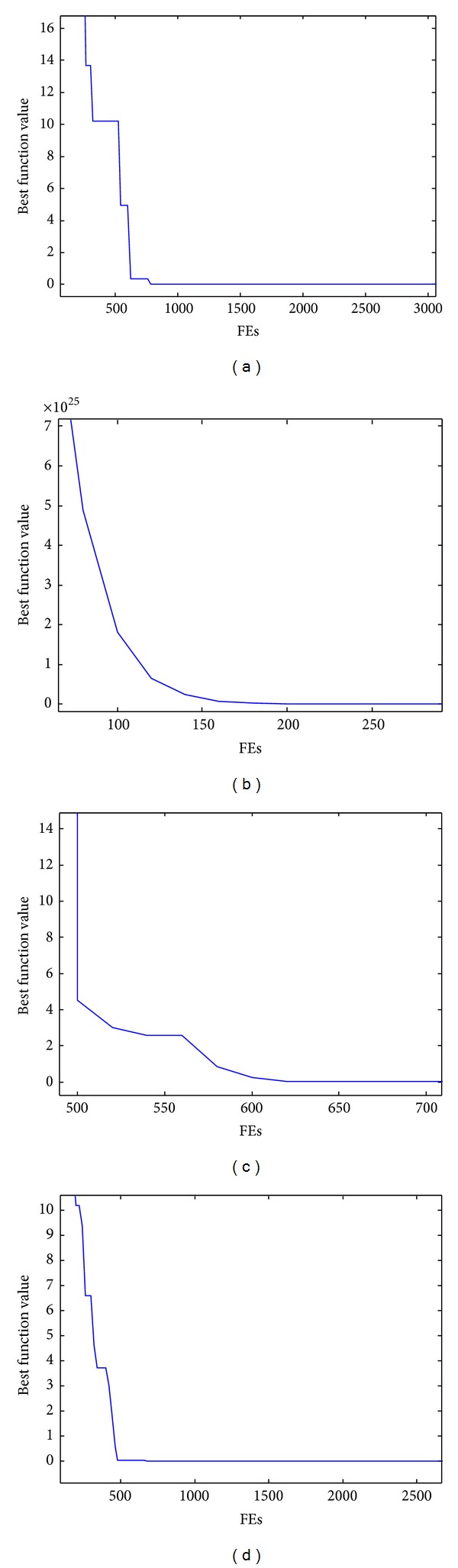
The evolutionary process of PSO-VRS on test functions, (a) Rosenbrock function, (b) variably dimensioned function, (c) Wood function, and (d) Ackley function.

To solve each test optimization function, each algorithm was run 30 times. The corresponding computational results are listed in Table 1, where Mean is the mean fitness value of 30 runs; Std is the related standard deviation of the results; FEs is the mean function evaluations to obtain the results. The evolutionary process of the best fitness value of each function obtained by PSO-VRS is displayed in Figure 2. 

From the results about the Rosenbrock function in Table 1, we can find that it is generally difficult for other PSO variants to find an optimal or near-optimal solution of the Rosenbrock function. However, with the aid of VRS, PSO-VRS can use the basic PSO to efficiently find the optimal solution averagely within about 12000 fitness evaluations. Moreover, Figure 2(a) demonstrates that VRS enables PSO to converge to high-quality solutions quickly.

Results of the variably dimensioned function listed in Table 1 reveal that it is hard for most of the comparative PSO variants without the integration of VRS to generate a solution of high quality. In contrast, PSO-VRS always produces the optimal solution in relatively small number of function evaluations (averagely about 12684).  Figure 2(b) demonstrates that PSO-VRS converges to the optimal solution quickly. The variably dimensioned function is highly related, which brings a great deal of difficulty to typical PSO variants to form satisfactory solutions. On the other hand, VRS utilizes the underlying variable relations and translates the original problem into a one-variable optimization task. This indicates that VRS significantly reduces the complexity of the variably dimensioned function. 

We can see from Table 1 that, compared with other comparative PSO variants, PSO-VRS generates the best result (also the optimal) of the Wood function averagely within no more than 13100 function evaluations. Though the variable relations described in (17)–(20) seem more complex, they well support PSO to find the optimal solution.  Figure 2(c) underlines that PSO-VRS exhibits high convergence.

It also can be observed from Table 1 that both CLPSO and PSO-VRS could always find the best results, compared to other PSO variants. The superiority of PSO-VRS to CLPSO is that PSO-VRS can obtain the optimal solution at the cost of much less function evaluations. The high convergence capability of PSO-VRS when solving the Ackley function is shown in Figure 2(d). 

## 4. Experimental Study on a Real-World Optimization Problem

Frequency-modulated (FM) sound wave synthesis has an important role in several modern music systems and to optimize the parameter of an FM synthesizer is a six-dimensional optimization problem where the vector to be optimized is represented by *X* = {*a*_1_, *ω*_1_, *a*_2_, *ω*_2_, *a*_3_, *ω*_3_} [22, 23]. This problem is a highly complex multimodal one having strong epistasis with minimum value *f*(X*) = 0 [24]. This problem was frequently solved by EAs or taken as a benchmark real-world optimization problem to test the performance of new EA variants [25, 26]. This optimization problem is formulated as follows [22]:

(23) y(t)=a1·sin(ω1·t·θ    +a2·sin(ω2·t·θ+a3·sin(ω3·t·θ))),y0(t)=(1.0)·sin((5.0)·t·θ−(1.5)   ·sin((4.8)·t·θ+(2.0)·sin((4.9)·t·θ))).

The objective function is

(24) $$\begin{array}{c} \min f\left(X\right)=\overset{100}{\underset{t=0}{\sum }}{\left(y\left(t\right)-y_{0}\left(t\right)\right)}^{2}. \end{array}$$

To use the variable reduction strategy, we let the derivative of the objective function on variable *a*_1_ equal to zero and obtain

(25) $$\begin{array}{c} \overset{100}{\underset{t=0}{\sum }}2\cdot \left(a_{1}\cdot \phi \left(t\right)-y_{0}\left(t\right)\right)\cdot \phi \left(t\right)=0, \end{array}$$

where *y*(*t*) = *a*_1_ · *ϕ*(*t*), *ϕ*(*t*) = sin(*ω*_1_ · *t* · *θ* + *a*_2_ · sin(*ω*_2_ · *t* · *θ* + *a*_3_ · sin(*ω*_3_ · *t* · *θ*))). 

From (25), we have

(26) $$\begin{array}{c} a_{1}=\frac{\sum_{t=0}^{100}y_{0}\left(t\right)\cdot \phi \left(t\right)}{\sum_{t=0}^{100}\phi \left(t\right)\cdot \phi \left(t\right)}. \end{array}$$

According to (26), variable *a*_1_ can be calculated from other five variables. Therefore, *a*_1_ is the reduced variable and collection {*ω*_1_, *a*_2_, *ω*_2_, *a*_3_, *ω*_3_} is the corresponding optimization variable core. With VRS, the solution space is shrunk from six dimensions to five dimensions. 

To evaluate the impact of VRS on this optimization problem, we use PSO-w, PSO-cf, UPSO, FDR, FIPS, CPSO-H, and CLPSO with and without the integration of VRS to solve the problem, respectively. Each algorithm is run for 30 times. 

From Table 2, we can discover that, for every PSO variant, the results produced by the algorithm with the integration of VRS are better than that produced by the algorithm without the integration of VRS. Particularly, this improvement is more significant when FIPS and CLPSO are taken as the solvers. These results demonstrate the potential application of VRS to real-world optimization problems. Moreover, in this optimization problem, only one variable is reduced, which indicates that when we apply VRS to optimization problems, we do not have to find all the quantitative variable relations and reduce major variables, even the reduction of small number of variables could be beneficial and improve the efficiency of EAs.

## 5. Conclusions

The utilization of the domain knowledge associated with the optimization problem can reduce the complexity of the original problem and facilitate the solution search of EAs. In this study, we investigate the underlying knowledge of quantitative variable relations that have to be satisfied in the optimal solutions of an unconstrained and first-order derivative optimization function. Based on these relations, we propose a variable reduction strategy (VRS). The essence of VRS is to find an optimization variable core with the minimum number of core variables. Computational results and comparative studies carried out for several test benchmark optimization functions and a real-life optimization problem demonstrate that VRS can significantly improve the efficiency of PSO variants. Currently, we cannot guarantee that VRS can be applied to any unconstrained optimization problems. However, it could be beneficial to check the variable relations and use VRS when using EAs to solve unconstrained optimization problems. VRS is expected to have large application potential in real-world optimization problems.

Some future researches can be carried in four directions. Although the variable reduction strategy is generic and effective, on some occasions, it might be very difficult to obtain the variable relations from the partial derivatives of an optimization function because of the complexity of these derivatives. To construct a solid and comprehensive theory of variable reduction, we may investigate whether there are some generic and formal theories about finding the explicit variable relations through a group of equations. Furthermore, we also consider formulating the underlying variable relations by some approximate methods, such as neural network. The third direction of research is to further study the variable reduction strategy to apply it to constrained optimization problems. The fourth one is to test the efficiency and effectiveness of the variable reduction strategy with regard to more real-world optimization problems. 

## Figures and Tables

**Table 1:** Optimization results obtained for the test functions; the best results are shown in boldface.

Algorithm	Mean	Std	FEs	Mean	Std	FEs
	Rosenbrock function			Variably dimensioned function		
PSO-w	2.182*e* + 000	2.642*e* − 002	30 000	1.576*e* + 003	4.610*e* + 002	200 000
PSO-cf	3.833*e* − 001	0.250*e* + 000	30 000	5.618*e* + 000	1.385*e* + 001	200 000
UPSO	1.055*e* + 000	1.542*e* + 000	30 000	1.164*e* + 003	4.763*e* + 002	200 000
FDR	6.480*e* − 001	6.846*e* − 001	30 000	3.188*e* − 001	5.820*e* − 001	200 000
FIPS	1.186*e* + 000	1.080*e* − 001	30 000	4.479*e* + 003	1.317*e* + 003	200 000
CPSO-H	1.044*e* + 000	1.265*e* + 000	30 000	1.365*e* + 004	3.277*e* + 003	200 000
CLPSO	1.262*e* + 000	9.658*e* − 001	30 000	2.889*e* + 003	6.235*e* + 002	200 000
PSO-VRS	**0.0**	**0.0**	**11 565**	**0.0**	**0.0**	**12 684**

Algorithm	Wood function			Ackley function		

PSO-w	3.347*e* − 006	3.383*e* − 006	200 000	3.942*e* − 014	1.123*e* + 000	200 000
PSO-cf	9.784*e* − 006	2.225*e* − 007	200 000	1.123*e* + 000	8.655*e* − 001	200 000
UPSO	1.459*e* − 009	5.144*e* − 009	200 000	1.225*e* − 015	3.162*e* − 015	200 000
FDR	1.576*e* − 009	1.589*e* − 009	200 000	2.844*e* − 014	4.107*e* − 015	200 000
FIPS	5.868*e* − 005	7.834*e* − 005	200 000	4.812*e* − 007	9.172*e* − 008	200 000
CPSO-H	5.861*e* − 001	2.616*e* − 001	200 000	4.931*e* − 014	1.104*e* − 014	200 000
CLPSO	1.570*e* − 002	3.330*e* − 002	200 000	0.0	0.0	180 864
PSO-VRS	**0.0**	**0.0**	**13 078**	**0.0**	**0.0**	**11 254**

**Table 2:** Optimization results obtained for the frequency-modulated synthesizer optimization problem; the results obtained by EAs with VRS are shown in boldface.

Algorithm	Best	Worst	Mean	Std
PSO-w	0.0	1.258023*e* + 001	3.752319*e* + 000	5.853543*e* + 000
PSO-w-VRS	**0.0**	1.254014**e** + 001	3.375620**e** + 000	5.893543**e** + 000
PSO-cf	2.673488*e* − 006	2.180154*e* + 001	1.200588*e* + 001	5.144827*e* + 000
PSO-cf-VRS	4.275136**e** − 007	1.217917**e** + 001	8.466443**e** + 000	4.848450**e** + 000
UPSO	5.839747*e* − 028	1.473172*e* + 001	6.608215*e* + 000	4.636487*e* + 000
UPSO-VRS	**0.0**	1.120719**e** + 001	6.425027**e** + 000	4.185174**e** + 000
FDR	0.0	2.016705*e* + 001	1.191916*e* + 001	6.193037*e* + 000
FDR-VRS	**0.0**	1.254068**e** + 001	1.055711**e** + 001	3.626518**e** + 000
FIPS	3.321497*e* − 003	8.500601*e* + 000	2.306348*e* + 000	2.513825*e* + 000
FIPS-VRS	9.388098**e** − 010	2.787766**e** + 000	1.127794**e** + 000	1.359315**e** + 000
CPSO-H	1.179023*e* + 001	2.553845*e* + 001	1.910668*e* + 001	4.381173*e* + 000
CPSO-H-VRS	7.783981**e** − 001	1.683571**e** + 001	1.274518**e** + 001	5.058687**e** + 000
CLPSO	1.354023*e* − 004	1.138270*e* + 001	1.666438*e* + 000	3.567321*e* + 000
CLPSO-VRS	2.309327**e** − 005	6.541305**e** + 000	3.734962**e** − 001	1.456388**e** + 000
